# Meibomian Gland Outcome Measures in Dry Eye Treatment Trials

**DOI:** 10.3390/jcm15114093

**Published:** 2026-05-25

**Authors:** Shora M. Ansari, Jay Ruzhang Jiang, Andrew Loc Nguyen, Jerry R. Paugh

**Affiliations:** 1 Southern California College of Optometry, Marshall B. Ketchum University, Fullerton, CA 92831, USA; sansari@ketchum.edu; 2 Independent Researcher, Pico Rivera, CA 90660, USA; jiangrz02@hotmail.com; 3 Department of Mathematics, California State University, Fullerton, CA 90802, USA; anguyen@fullerton.edu

**Keywords:** meibomian gland dysfunction, meibomian glands, grading scales, meibum, dry eye, outcome measures, test efficacy, repeatability, reproducibility

## Abstract

**Background/Objectives**: This review considered meibomian gland functional assays, reported in recent dry eye treatment trials, that have potential as a clinical/physiological measure in a Core Outcome Set in dry eye clinical trials. The focus was on clinical methods that can be applied globally by trained clinicians. **Methods**: An electronic search of the MEDLINE database (via PubMed) was conducted to identify randomized controlled trials published in English between 1 January 1995 and 11 December 2025. The search strategy used the terms (*meibomian OR meibography*) *AND treatment*. Studies were included if they reported outcomes related to meibum secretion and/or meibomian gland expressibility in human participants. Moreover, a retrospective chart review was undertaken from previously unpublished meibum grading data to determine whether opacity and viscosity grades for meibum are correlated. **Results**: 98 studies were included for analysis The grading systems of Bron and co-workers, combined with that of the similar MGD Workshop report, were the most prevalent (*n* = 48), followed by Lane and co-workers (*n* = 23) and Shimazaki and co-workers (*n* = 10). Expressibility grading systems were most prevalent for Pflugfelder et al. (*n* = 33), followed by Blackie-Korb (*n* = 19) and Author defined (*n* = 17). The retrospective analysis of 69 charts suggests high correlation between meibum opacity and viscosity (Pearson’s r = 0.904, *p* < 0.001. 95% CI 0.849–0.940). **Conclusions**: Grading meibum quality is important in dry eye diagnosis and treatment. A modification of the MGD Workshop system appears useful as a core outcome set parameter in dry eye treatment trials.

## 1. Introduction

The primary pathophysiology of obstructive meibomian gland disease is blockage of the terminal gland ducts that in turn limits the physiological excretion of meibum onto the ocular surface. Excretion inhibition leads to meibum stagnation, increased pressure within the gland, and eventual acinar atrophy [1,2].

Clinical assessment of human meibomian gland function involves characterization of meibum appearance, expressibility and indirectly the thickness and appearance of the lipid layer on the ocular surface. “Expressibility” has been characterized as both the amount of pressure required to express meibum [3] and the number of glands providing meibum [4,5]. Anecdotally, most clinicians seem to use digital expression to grade meibum color, viscosity and ease of expression to identify MGD as a component of dry eye.

Early research reports of meibomian gland function centered around digital expression of the lower and upper meibomian glands; e.g., McCulley and Sciallis [6], Korb and Henriquez [7], Norn [8] and Shimazaki et al. [3]. In a series of blepharitis patients, McCulley and Sciallis [6] demonstrated the importance of meibum delivery to the ocular surface by demonstrating the increase in tear stability to normal or supranormal levels with deep gland expression.

More recent dry eye therapeutic studies have used digital [9], a controlled pressure device (Meibomian Gland Evaluator, MGE) [10] and cotton-tipped applicators [11] to assess meibomian gland treatment efficacy. The issue in current and future dry eye treatment trials is that several systems for assessing meibomian gland function have been reported, with no consensus as to which systems and parameters are important to reliably determine therapeutic outcomes.

Core outcome sets (COSs) are being developed for dry eye treatment trials that can standardize the conduct of the trial [12,13]. A COS for dry eye involves consideration of patient symptoms and quality of life [13] but also comprises Physiological/Clinical domains [14] that must employ reliable protocols to assess important disease parameters. To the best of our knowledge, the outcome set parameters in dry eye management, beyond patient-centric outcomes, have yet to be determined.

Employing a COS for dry treatment trials would allow direct comparison of therapeutic efficacy studies and systematic reviews and meta-analyses of a given management approach. Given the exponential growth in dry eye treatments since the early 2000s, and in particular the growing number of therapies aimed at ameliorating MGD, it seems timely to consider which grading systems for meibomian gland function are the most important. The grading system must be efficacious in dry eye diagnosis, repeatable and reproducible for use in multi-center investigations.

The aim of this review is to consider the various meibomian gland functional assays reported in the most recent decades of dry eye treatment trials. We focus on clinical methods that can be applied globally by trained clinicians, excluding the several methods that require custom or specialized equipment such as tear film interferometers.

## 2. Materials and Methods

### 2.1. Literature Survey

An electronic search of the MEDLINE database (via PubMed) was conducted to identify randomized controlled trials published in English between 1 January 1995 and 11 December 2025. The search strategy used the terms (*meibomian OR meibography*) *AND treatment*. Studies were included if they reported outcomes related to meibum secretion and/or meibomian gland expressibility in human participants.

All identified records were imported into Covidence (Veritas Health Innovation, Melbourne, Australia; available at covidence.org) for systematic screening. Titles and abstracts were reviewed to assess eligibility based on predefined inclusion criteria: reports had to be in English, were randomized controlled treatment trials and evaluated meibomian gland function.

Full-text articles of potentially relevant studies were subsequently retrieved and evaluated, with reasons for exclusion documented at this stage. The reference lists of included studies were also manually reviewed to identify any additional relevant articles that may have been missed during the initial search.

**Figure 1:**
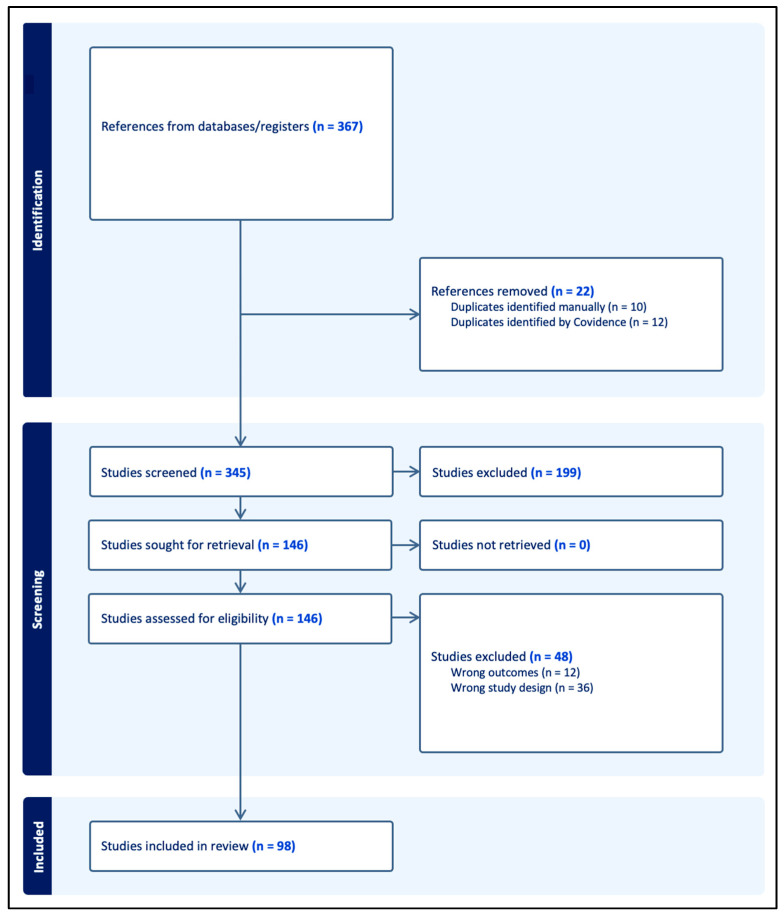
PRISMA-ScR flow diagram. Note: at the initial screening step (*n* = 345), 199 articles were excluded for non-adherence to review inclusion criteria.

The study selection process is summarized in Figure 1 using the Preferred Reporting Items for Systematic Reviews and Meta-Analyses extension for Scoping Reviews (PRISMA-ScR) flow diagram. From the included studies, data were extracted on sample size, study population, intervention and comparator, as well as methods used to grade meibum secretion and meibomian gland expressibility.

### 2.2. Retrospective Chart Review

Along with review of meibum grading methods (e.g., type and amount of pressure applied), we considered the descriptors used in the several grading systems. Is appearance alone sufficient to grade the meibum: e.g., opacity, or color, or both combined? Can the appearance grade be enhanced by assessing relative viscosity; e.g., “inspissated (toothpaste)” used in several scales [15,16] (Table 1)?

As part of a human study to characterize meibum using hyperspectral stimulated Raman spectroscopy [21], detailed meibum grading descriptions and data from approximately 130 charts were available, heretofore unpublished. This study was conducted from November 2014 to June 2017 at the Southern California College of Optometry and the Department of Ophthalmology, University of California Irvine.

The grading scales employed were novel to the Raman study and developed by two experienced MGD investigators (JVJ and JRP) [21]. All grading was undertaken by a single investigator (JRP). The detailed grading scales for opacity and viscosity were as follows:

**Opacity scale**: 0 = clear, 1 = partial obscuration of margin detail, 2 = mostly obscures margin detail, 3 = opaque

**Viscosity:** 0 = olive oil, 1 = slow spreading, 2 = very slow spreading, like corn syrup, 3 = toothpaste

We also used the Bron system grading [15] to assign an overall grade to the available meibum. All three scales were judged by the investigator using fine scale increments (0.1) that have been demonstrated to allow the clinician a greater ability to detect change in subjective scales [22].

Up to 31 charts were arbitrarily selected from subjects designated as primarily normal (non-dry eye), aqueous tear deficient, or as having MGD. The dry eye classification was based on fluorescein breakup time (<6.0 s considered dry [23]), NEI staining (greater than 6.0 considered dry [24,25]) and Schirmer I without anesthesia, strips re-positioned at 2.0 min if no wetting shown [26] (dry less than 5.0 mm wetting at 5 min). Detailed test descriptions are found in the initial Raman report [21].

**Figure 2:**
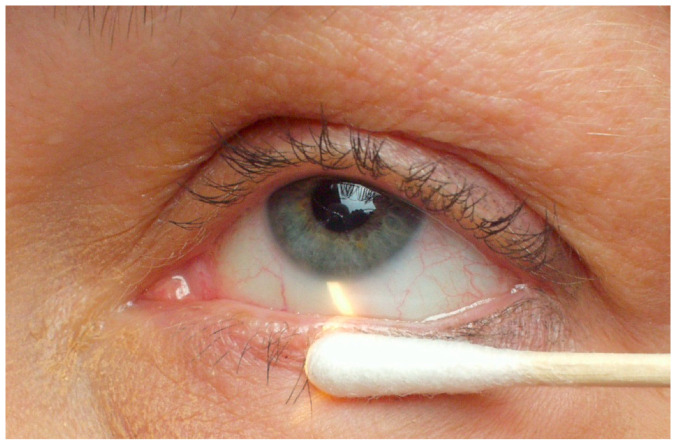
Tangential gland expression; meibum grade reported as the average across the entire lower eyelid, 0–3 scale.

The meibomian secretion evaluation was accomplished using a wooden handle cotton-tipped applicator, applied with gentle pressure for 5–10 s, in a tangential manner to each lower eyelid margin (Figure 2). The grades as above were the average appearance of each lower eyelid for as many glands as provided visible meibum. Only data from the lower eyelid, belonging to the worst eye in terms of NEI staining, were used for the present analysis.

The opacity and opacity grades were extracted for each subject, along with the Bron grade [15]. Pearson analysis was undertaken to determine correlation between the opacity and viscosity grades and for viscosity and Bron grades. ANOVA and Dunnetts test were undertaken to compare Bron scale grades among the normals, aqueous tear deficient (ATD) and Meibomian Gland Dysfunction (MGD) subjects.

In addition to the scale grading, the investigator recorded descriptions of the meibum appearance; e.g., “clear with particles”, “cloudy”, “inspissated”, “yellow”, or “white”, or mixtures of these descriptors within the meibum.

## 3. Results

### 3.1. General Results

Major systems to assess meibomian gland function in clinical trials are summarized in Table 1. Lipid layer interferometry for thickness and appearance was not included since lipid characteristics are an indirect measure of meibum delivery to the ocular surface. We are concerned with identifying measures of MG function that should be a physiological/clinical component in a dry eye COS.

The two major functional tests for meibomian gland health are expressibility, meaning the number of glands expressing, and assessing the quality of the gland excreta (meibum). Prior to the MGD Workshop reports [19,27], expressibility and meibum quality were largely used separately in clinical trials but more recently have been used concurrently in treatment investigations (e.g., see Lane et al. [20] and Eom et al. [28]). Surprisingly, we could find little to no formal test efficacy data for these various functional systems, nor could we find information regarding repeatability nor inter-observer variability.

### 3.2. Therapeutic Studies, 1995 to Present

A total of 98 studies matched the search criteria for clinical trials involving assessment of meibomian gland function. Table 2 lists the number of systems used to grade meibum quality, and Table 3 demonstrates the systems used for gland expressibility.

The most prevalent meibum grading system (27 instances) was that of Bron and co-workers, first reported in 1991 [15], and the similar (for meibum grading) MGD Workshop Management and Treatment report (21 instances; Table 4, Geerling et al. [19]). The meibum grading system promulgated by Lane et al. [20] was reported in 23 clinical trials, followed by that of Shimazaki et al. [17]. Overall, where the systems could be understood, 84 of the 98 included reports employed a system to grade meibum quality.

The most prevalent meibomian gland expressibility system was that of Pflugfelder et al. [4], 33 reports, followed by Blackie-Korb [18] (19 reports) and Author defined. Overall, 68 studies reported some measure of expressibility (number of glands expressing liquid excreta).

### 3.3. Retrospective Chart Review

#### 3.3.1. General Results, Meibum Grades and Descriptions

The raw data from the retrospective chart review are available in Supplementary Material, Table S1, Retrospective chart review data.

Thirty-one clinically normal (non-dry eye), 31 MGD and 12 ATD subjects provided Bron scale grades and investigator descriptions of the meibum. Sixty-nine of these had data for the correlational analysis. The mean and SD for the opacity, viscosity and Bron grades are listed in Table 4. For all Bron grades, both opacity and viscosity grades were within 0.5 scale unit increments of the Bron assessment.

**Table 4:** Correlational analysis, retrospective chart data *.

Sample	Opacity Grade ^†^ Mean (SD)	Viscosity Grade ^†^ Mean (SD)	Bron Grade ^†^ Mean (SD)	Correlation ^‡^: Opacity–Viscosity Scale (95% CI)	Correlation ^‡^: Viscosity–Bron Scale (95% CI)
Normal *n* = 31	1.0 (0.4)	1.0 (0.3)	1.1 (0.4)	r = 0.770, *p* < 0.001 (0.572–0.883)	r = 0.857, *p* < 0.001 (0.722–0.929)
Aqueous Deficient *n* = 7	1.2 (0.6)	1.2 (0.8)	1.6 (0.6)	r = 0.911, *p* = 0.004 (0.505–0.987)	r = 0.787, *p* = 0.036 (0.085–0.967)
MGD *n* = 31	1.4 (0.7)	1.3 (0.7)	1.4 (0.6)	r = 0.952, *p* < 0.001 (0.903–0.977	r = 0.915, *p* < 0.001 (0.830–0.959)
Combined *n* = 69	1.2 (0.6)	1.2 (0.5)	1.3 (0.6)	r = 0.904, *p* < 0.001 (0.849–0.940)	r = 0.869, *p* < 0.001 (0.796–0.917)

* from the study of Paugh et al., 2019 [21], ^†^ scale 0–3; refer to text, ^‡^ Pearsons r.

In the normal sample, 25 of the subjects had Bron grades (0–3 scale) of 1.0 or less, considered the diagnostic level for MGD [29]. Conversely, 6 of the normals had a Bron scale grade of greater than 1.0 (data not shown). By investigator description, 21 of 31 subjects had meibum described as “clear”, with a majority (>50%) of the clear descriptions also describing “particles”.

The MGD sample yielded 29 of 31 subjects who demonstrated ≥1.0 Bron grades, with 2 having grades <1.0. Of those with Bron grade >1.0, 10 had meibum descriptions of “clear with particles”, with the remaining 19 having descriptions of cloudy and inspissated and mixtures of same.

The ATD sample had all Bron grades ≥1.0 (*n* = 12) with mixed descriptions; e.g., “clear, particles, opaque, inspissated”, “clear, many particles”.

#### 3.3.2. Correlational and Statistical Analysis

**Figure 3:**
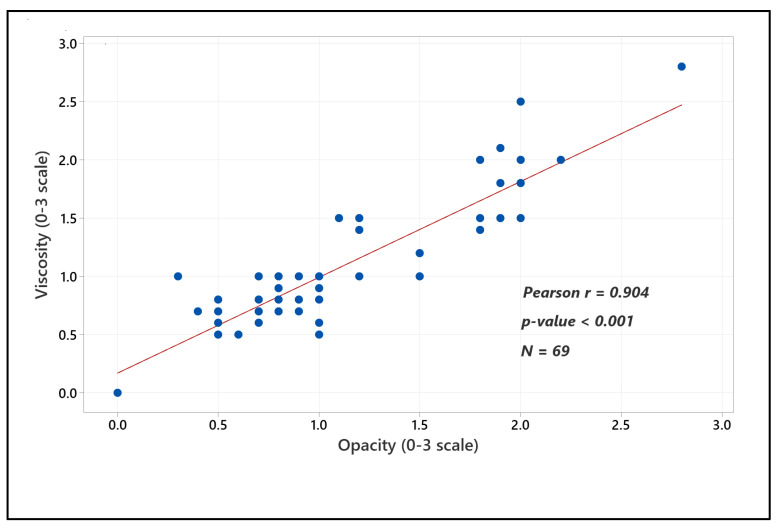
Opacity–viscosity correlations. Data from study of Paugh et al. [21]; the raw data supporting this figure can be found in the Supplementary Materials, Table S1, Retrospective chart review data.

The summary data and correlational analyses are listed in Table 4. Both the individual dry eye subtype and the overall separate opacity–viscosity (*n* = 69) correlations demonstrated good to excellent correlation and highly statistically significant associations. The Bron scale (Bron et al., 1991 [15]) is primarily an opacity scale and was also highly correlated with the viscosity grades for the sample (*n* = 64; five individual viscosity grades were unavailable for the ATD group); Table 4, Figure 3.

Among-group comparisons for Bron scale grades demonstrated increasing values (0–3 scale) to aqueous tear deficients from normals (Table 4). Only the normal to MGD comparison achieved statistical significance (Dunnetts test, difference of means 0.316, 95% CI (−0.002–0.634, *p* = 0.05).

## 4. Discussion

Delivery of meibum to the ocular surface is critical to the management of meibomian gland dysfunction, evidenced by the myriad MGD treatments that ultimately aim to unblock functioning glands to allow meibum to spread to the tear film. Digital meibum expression stabilizes the tear film in blepharitis [6], thickens the tear lipid layer and stabilizes the tear film [30] and reduces evaporation in normal and MGD subtypes [31]. It is clear that expressibility of the meibomian glands (number of glands expressing meibum) and meibum quality are key indicators for diagnosis and treatment trials in MGD.

Lack of gland expressibility occurs when the orifices are capped or plugged or the gland has atrophied (individual glands are missing). Expressibility, the number of glands expressing liquid secretion, has been quantified for 5 central glands of the lower eyelid [4] and the entire lower eyelid [5]. This measure provides some indication of meibomian gland function. However, reduced expressibility can be remedied when the underlying gland is present and retains some function.

Treatments such as debridement scaling [32,33] and keratin solubilization using selenium sulfide [34] have demonstrated efficacy in freeing meibomian gland secretion. Because poor expressibilty can be remedied, expressibility per se is a lesser indicator of meibomian gland function or dysfunction. In contrast, meibum *quality*, that is useful in dry eye diagnosis and improves with a broad array of therapeutic treatments, should be a key clinical/physiological parameter in a core outcome set for dry eye clinical trials.

Since 1995, several recurring systems have been used to characterize meibum quality in clinical trials (Table 1). Chronologically, these are Bron et al., 1991 [15], Mathers et al. [16], Shimazaki et al., 1998 [17], Geerling et al., 2011, Table 4 [19] and Lane et al., 2012 [20]. The systems of Bron et al., 1991 [15] and Geerling et al., 2011 [19] can be grouped, as they have similar descriptions of the meibum quality, although the MGD Workshop approach specified the lower eyelid and central 8 glands to provide a 0–24 scale. For characterization purposes, we refer to this system as the “MGD Workshop” system.

We found a majority of the dry eye treatment trials (*n* = 48) used the Bron et al. [15]—MGD Workshop report [19] system, followed at a lower frequency (*n* = 23) by Lane et al. [20], Shimazaki et al. [17] and Mathers et al. [16] (Table 2). Some reports used slight variation of the original systems; e.g., Zhang et al. [35] used the MGD Workshop grading system, but only excreta of five glands were graded vs. the 8 suggested in the MGD Workshop. Other variabilities included assessing the upper vs. lower eyelids, the amount and method to apply pressure to the eyelid margin and the meibum quality descriptions.

Given the importance of accurately assessing meibum quality for both dry eye diagnosis and treatment, standardization of a reliable method is critical.

### 4.1. General Considerations of a System to Evaluate Meibum Quality

An effective system of assessing meibum must be clinically valid (i.e., the grade is accurate for health or disease and worsens with greater compromise of the function), repeatable under the same investigator, patient and ambient conditions (intraobserver agreement) and reproducible (data agree if conducted by differing investigators of similar patients in different locations). Review of the selected reports herein found little diagnostic test efficacy data for the various functional systems and also few reports of grading system repeatability or reproducibility. These requirements must be met for an effective system to evaluate meibomian gland function by the characterization of meibum.

### 4.2. Meibum Characterization

Review of the reports of the various grading systems (Table 1) suggests some level of opacity and viscosity designation for the meibum grades from normal to greater compromise, particularly for Grade 3 (described as inspissated, like toothpaste [15,16,19,20]). Moreover, the retrospective chart review, reported herein, where separate viscosity and opacity scales were proposed, demonstrates a high correlation between these two aspects of meibum obtained under gentle pressure (Figure 3). Developing a grading scale combining degree of opacity concurrent with viscosity appears useful.

### 4.3. Lid Margin Applanation Pressure

Korb and Henriquez [7] were perhaps the first group to differentiate gentle vs. forceful (with eyelid backing) expression of meibomian glands; they found increases in the percentage of secreting glands with forceful expression. Subsequently, Korb and Blackie [5] developed a constant pressure device that applied gentle pressure (1.25 g/mm^2^) to the eyelid margin that was equivalent to the pressure of a forced blink. The device has become commercialized as the Meibomian Gland Evaluator (MGE, Johnson and Johnson Vision care, Jacksonville, FL, USA) and used in many diagnostic and treatment clinical trials.

The concept of applying pressure equivalent to a forceful blink to grade secretions is logical since it represents the pressure exerted on the meibomian glands under physiological conditions. If no secretions can be elicited under gentle pressure, the gland may be missing, the central duct blocked or the orifice capped. As an alternative to using the MGE, gentle pressure applied using a cotton-tipped applicator (CTA) has been demonstrated to be useful in dry eye diagnosis [25] and in a therapeutic trial [11].

Using the CTA for routine meibum grading, anecdotal responses from patients at our institution suggest that the pressure is noticeable but not painful (unpublished data). More formal subjective discomfort ratings could be achieved using a Numerical Rating scale [36,37] during the expression. Objective (using a pressure transducer) pressure data could be generated in vivo [38] and ex vivo using a mock eyelid apparatus to determine whether the pressure of a CTA approaches that of a deliberate blink [5].

### 4.4. Number of Glands Assessed and Location

Blackie and Korb [18] first suggested assessing three sectors of the lower eyelid, for five consecutive glands each, for meibomian glands yielding liquid secretion MGYLS, (expressibility). Lane et al. [20] recorded MGYLS but also assessment of meibum quality in the 15 glands of the lower eyelid. More recent therapeutic studies have adopted the Lane et al. [20] approach (e.g., Schanzlin et al. [39], Watson et al. [34]).

Assessing 15 glands of each lower eyelid, using a 0–3 scale for each gland, provides a semi-continuous scale (0–45), useful for diagnosis and measuring change in a clinical trial. However, it seems challenging to accurately record five meibum grades concurrently, even when having a scribe record the findings. In contrast, using the central 8 glands of the lower eyelid (MGD Workshop method [19]), appears more efficient compared to 15 glands assessed yet effective in monitoring change in clinical trials [40,41,42].

### 4.5. Scale Unit Increments

Most scales to grade meibum are integer scales, 0–3 or 0–4, delineated by descriptions of opacity and/or viscosity. Bailey et al. [22] have demonstrated the utility of finer scale increments to detect change using subjective scales. Efron et al. [43] have applied finer scale increments to real-world clinician grading for corneal staining, conjunctival redness and papillary hypertrophy using standard image scales. Despite instructions to grade using 0.1 scale unit increments, the graders clustered the grades around integer and 0.5 scale unit increments on 0–4 scales [43]. It seems reasonable to try to grade meibum quality in 0.5 scale unit increments.

### 4.6. Summary and Proposed Scale

Based on the evidence summarized herein, grading meibum quality is a useful and desirable parameter in dry eye therapeutic clinical trials and should be one component of a core outcome set. Meibum quality demonstrates compromise in dry eye disease and improvement with effective therapeusis. What remains is consensus as to an effective system to grade meibum quality.

The MGD Workshop system to grade meibum (Table 4, Geerling et al. [19], is one of the most widely reported systems, has been recommended to assign MGD severity [44] and appears to be simple and efficient to apply in clinical trials. We believe that the MGD Workshop system can achieve greater usefulness/utility by specifying the method of gentle pressure application, refining the grading descriptions and incorporating finer (0.5) scale unit increments.

We propose the following approach, titled “Modified MGD Workshop Meibum Grading System”.

#### 4.6.1. Meibum Grading System Overview

Detailed rationale and methods can be found in the Supplementary Materials, Protocol, Modified MGD Workshop Meibum Grading System.

**Figure 4:**
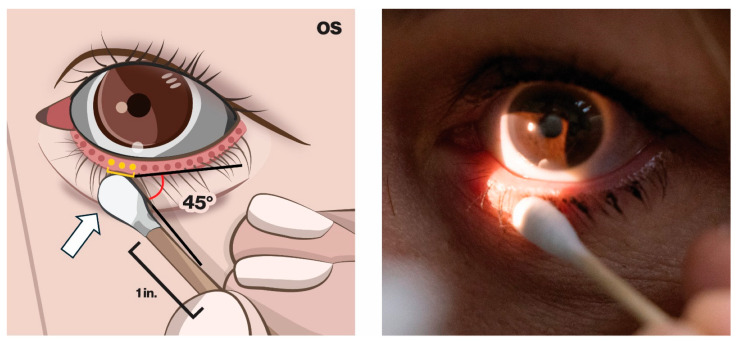
Demonstration of expression technique, wooden-handle cotton-tipped applicator, gentle pressure applied, 45-degree angle to eyelid margin, hold for 5–10 s, observe at 16×.

- The central 8 glands of the lower eyelid of each eye are examined, with an anchor at the 6:00 position of the cornea and 3–4 glands on either side assessed.
- The eyelid margin is first cleaned of debris using a saline-wetted cotton-tipped applicator (CTA).
- A fresh sterile, wooden-handle cotton-tipped applicator is used at a 45-degree angle to the eyelid margin and applied using gentle pressure (patient does not report pain or troublesome irritation); Figure 4.
- The pressure is maintained for 5–10 s, attempting to evert the margin slightly to expose the orifices to grade the meibum.
- Approximately three glands/orifices are graded concurrently (see proposed opacity– viscosity scale below)
- The CTA is moved slightly nasally or temporally to evaluate the next three orifices.
- Each orifice is graded on the 0–4 scale, referring to the opacity–viscosity scale.
- A total scale range for each eyelid is 0–32, with greater numbers denoting greater compromise of meibomian gland function.
- Meibum grading is repeated for the fellow eye if a bilateral investigation.

#### 4.6.2. Proposed Scale for Grading Meibum

- 0 = clear, low viscosity, easily expressed, may have a few particles
- 0.5 = partially opaque, low viscosity, easily expressed, may have a few particles
- 1.0 = opaque, low viscosity, obscures margin details
- 1.5 = opaque, slightly elevated viscosity (greater than 1.0 but less than 2.0)
- 2.0 = opaque, increased viscosity, may extrude gel-like globules
- 2.5 = opaque, not completely inspissated
- 3.0 = opaque, inspissated, like toothpaste
- 3.5 = opaque, secretions retain form after expression (pouting [15])
- 4.0 = no excreta visible (due to blocked or missing gland)

Approximately three orifices are assessed at each CTA placement and recorded using the scale above. For each orifice, a grade of 0–4 is recorded, with a total score range of 0–32 per eyelid. Gland expressibility is recorded as “0” if no liquid excreta are observed or “1” if the gland does express liquid excreta. The total for each eyelid is 0–8: 8 if all 8 glands demonstrate liquid. The process is repeated for the fellow eye if desired.

### 4.7. Study Limitations and Future Directions

A limitation of this study was that the article sourcing was limited to the MEDLINE database. More comprehensive assessment of meibomian gland functional grading systems should be assayed using Embase, Web of Science and the Cochrane Library.

Modification of the MGD Workshop meibum grading system may provide an efficient, semi-quantitative and widely applicable method to standardize the evaluation of meibum in clinical trials. Preliminary information using this scale suggests moderate to excellent repeatability for single experienced investigators and moderate to good interobserver agreement [45]. A caveat to use of the new scale is that it will be less helpful in cases of meibomian seborrhea (i.e., high delivery) MGD [46]. In meibomian seborrhea, the meibum is of poor quality but generally clear and easily expressed [47].

Training in the proposed system will be essential. A series of still images of the various grades of meibum is needed, as is video of the various grades, labeled by consensus of expert examiners.

Future investigations must examine grading system test efficacy, repeatability and inter-observer variability. If the system for both expressibility and meibum quality are found to be efficacious in diagnosis and treatment, a consensus process should be convened among interested parties to determine whether, and which systems, should be incorporated into a core outcome set for clinical trials in dry eye [13].

## 5. Conclusions

Grading meibum quality is important in dry eye diagnosis and treatment. A modification of the MGD Workshop system may be useful to establish meibum quality as a core outcome set parameter in dry eye treatment trials.

## Figures and Tables

**Table 1:** Major meibomian gland function grading systems *.

Principal Authors	Technique	Functional Assay	Eyelid Region	Grading Scheme	Scale Type	Notes
Bron et al., 1991 [15]	Pressure unspecified; digital or glass rod	Appearance, viscosity of excreta	UL and LL	0: clear (i.e., normal) 1: cloudy; diffusely turbid 2: granular; turbid, fluid with particles 3: inspissated (toothpaste)	primarily opacity	Total scale: 0–3
Mathers et al., 1991 [16]	Firm digital pressure until excreta observed	Appearance, viscosity of excreta	LL all glands	1: Normal, clear, may have a few particles 2: Opaque, normal viscosity 3: Opaque, increased viscosity 4: Severe thickening (toothpaste)	opacity and viscosity	Total scale: 1–4
Shimazaki, et al., 1998 [17]	Variable digital pressure	Appearance, amount of pressure required	UL	0: clear, easily expressed 1: cloudy, mild pressure 2: cloudy, >moderate pressure 3: meibum not expressed with hard pressure	opacity and pressure	Total scale: 0–3
Pflugfelder et al., 1998 [4]	Digital compression	Expressibility of 5 glands	UL and LL	0: all glands expressible 1: 3–4 glands 2: 1–2 glands 3: no glands	# expressible	Total scale: 0–3
Blackie and Korb, 2010 [18]	Standardized pressure device	Expressibility, MGYLS ^†^	LL 3 sectors of 5 glands each	# of glands with liquid secretion	# expressible	First report using meibomian gland evaluator (MGE) Total scale: 0–5 for 5 glands
MGD Workshop report, 2011 [19]	Method not specified	Appearance, viscosity of excreta	LL 8 central glands	0: clear 1: cloudy; diffusely turbid 2: cloudy with debris (granular) 3: thick, like toothpaste	primarily opacity	Total scale: 0–24
MGD Workshop report, 2011 [19]	Method not specified	Expressibility of 5 glands	UL or LL	0: all glands expressible 1: 3–4 glands 2: 1–2 glands 3: no glands	# expressible	As Pflugfelder et al. Total scale: 0–3
Lane et al., 2012 [20]	Standardized pressure device	Appearance, viscosity of excreta	LL 15 glands	3: clear, liquid 2: cloudy, liquid 1: inspissated, toothpaste 0: no secretion	primarily opacity	5 consecutive glands in 3 sectors Total scale: 0–45
Lane et al., 2012 [20]	Standardized pressure device	Expressibility; MGYLS ^†^	LL 15 glands	# of glands secreting any liquid	# expressible	5 glands in 3 sectors Total scale: 0–15 scale
Jiang et al., 2025 [11]	Gentle pressure; CTA ^‡,^ 45 degree angle	Appearance, viscosity of excreta	LL 8 central glands	0: clear meibum 1: cloudy 2: cloudy with particles 3: inspissated (toothpaste) 4: no meibum	primarily opacity	Modified MGD Workshop: 3 orifices per pressure Assign each orifice 0–4 Total scale per eyelid 0–32
Jiang et al., 2025 [11]	Gentle pressure; CTA ^‡^, 45 degree angle	Expressibility of 5 central LL glands	LL 5 central glands	0: all glands expressible 1: 3–4 glands 2: 1–2 glands 3: no glands	# expressible	Modified MGD Workshop: 3 orifices per pressure Assign each orifice 0 or 1 Total scale per eyelid 0–3

* reported in one or more therapeutic study, ^†^ MGYLS is “Meibomian Glands Yielding Liquid Secretion”; # the number of glands expressing in a zone or entire eyelid, ^‡^ CTA: wooden handle cotton-tipped applicator.

**Table 2:** Meibum grading systems reported in RCTs.

Grading System	Number
Bron 1991 [15]	27
Lane et al. [20]	23
MGDWS [19]	21
Mathers 1991 [16]	3
Shimazaki [17]	10
Author defined	5

**Table 3:** Meibomian gland expressibility scales utilized in RCTs.

Grading Scale	Number
Blackie-Korb [18]	19
Pflugfelder [4]	33
Author defined	17

## Data Availability

Data for the Retrospective Chart Review are available for download, as above, under “Supplemental Materials”. The Protocol for the Modified MGD Workshop Meibum Grading is available, as above, under “Supplemental Materials”.

## Supplementary Materials

The following supporting information can be downloaded at: https://www.mdpi.com/article/10.3390/jcm15114093/s1: Table S1, Retrospective chart review data; Protocol Modified MGD Workshop Meibum Grading System.

## Abbreviations

The following abbreviations are used in this manuscript:

ATD	Aqueous Tear Deficiency
CTA	Wooden handle cotton tipped applicator
MGE	Meibomian Gland Evaluator
MGD	Meibomian gland dysfunction
MGYLS	Meibomian Glands Yielding Liquid Secretion

## References

[1] Gutgesell V.J., Stern G.A., Hood C.I. (1982). Histopathology of meibomian gland dysfunction. Am. J. Ophthalmol..

[2] Knop E., Knop N., Millar T., Obata H., Sullivan D.A. (2011). The International Workshop on Meibomian Gland Dysfunction: Report of the Subcommittee on Anatomy, Physiology, and Pathophysiology of the Meibomian Gland. Investig. Ophthalmol. Vis. Sci..

[3] Shimazaki J., Sakata M., Tsubota K. (1995). Ocular surface changes and discomfort in patients with Meibomian gland dysfunction. Arch. Ophthalmol..

[4] Pflugfelder S.C., Tseng S.C.G., Sanabria O., Kell H., Garcia C.G., Felix C., Fuer W., Reis B. (1998). Evaluation of subjective assessments and objective diagnostic tests for diagnosing tear-film disorders known to cause ocular irritation. Cornea.

[5] Korb D.R., Blackie C.A. (2008). Meibomian gland diagnostic expressibility: Correlation with dry eye symptoms and gland location. Cornea.

[6] McCulley J.P., Sciallis G.F. (1977). Meibomain keratoconjunctivitis. Am. J. Ophthalmol..

[7] Korb D.R., Henriquez A.S. (1980). Meibomian gland dysfunction and contact lens intolerance. J. Am. Optom. Assoc..

[8] Norn M.S. (1987). Expressibility of meibomian secretion: Relation to age, lipid precorneal film, scales, foam, hair and pigmentation. Acta Ophthalmol..

[9] Goto E., Monden Y., Takano Y., Mori A., Shimmura S., Shimazaki J., Tsubota K. (2002). Treatment of non-inflamed obstructive meibomian gland dysfunction by an infrared warm compression device. Br. J. Ophthalmol..

[10] Korb D.R., Blackie C.A. (2010). Restoration of meibomian gland functionality with novel thermodynamic treatment device—A case report. Cornea.

[11] Jiang J.R., Khankan R., Ridder W.H., Nguyen A.L., Paugh J.R. (2025). A pilot randomized controlled trial of topical androgen treatment in dry eye. Ocul. Surf..

[12] Saldanha I.J., Le J.T., Solomon S.D., Repka M.X., Akpek E.K., Li T. (2019). Choosing Core Outcomes for Use in Clinical Trials in Ophthalmology: Perspectives from Three Ophthalmology Outcomes Working Groups. Ophthalmology.

[13] Saldanha I.J., Petris R., Han G., Dickersin K., Akpek E.K. (2018). Research Questions and Outcomes Prioritized by Patients with Dry Eye. JAMA Ophthalmol..

[14] Harman N.L., Gorst S.L., Williamson P.R., Barnathan E.S., Baughman R.P., Judson M.A., Junk H., Kampstra N.A., Sullivan E.J., Victorson D.E. (2022). Identifying a core outcome set for pulmonary sarcoidosis research—The Foundation for Sarcoidosis Research—Sarcoidosis Clinical OUtcomes Taskforce (SCOUT). Sarcoidosis Vasc. Diffus. Lung Dis..

[15] Bron A.J., Benjamin L., Snibson G.R. (1991). Meibomian gland disease. Classification and grading of lid changes. Eye.

[16] Mathers W.D., Shields W.J., Sachdev M.S., Petroll W.M., Jester J.V. (1991). Meibomian gland dysfunction in chronic blepharitis. Cornea.

[17] Shimazaki J., Goto E., Ono M., Shimmura S., Tsubota K. (1998). Meibomian gland dysfunction in patients with Sjogren syndrome. Ophthalmology.

[18] Blackie C.A., Korb D.R. (2010). The diurnal secretory characteristics of individual meibomian glands. Cornea.

[19] Geerling G., Tauber J., Baudouin C., Goto E., Matsumoto Y., O’Brien T., Rolando M., Tsubota K., Nichols K.K. (2011). The International Workshop on Meibomian Gland Dysfunction: Report of the Subcommittee on Management and Treatment of Meibomian Gland Dysfunction. Investig. Ophthalmol. Vis. Sci..

[20] Lane S.S., DuBiner H.B., Epstein R.J., Ernest P.H., Greiner J.V., Hardten D.R., Holland E.J., Lemp M.A., McDonald J.E., Silbert D.I. (2012). A new system, the LipiFlow, for the treatment of meibomian gland dysfunction. Cornea.

[21] Paugh J.R., Alfonso-Garcia A., Nguyen A.L., Suhalim J.L., Farid M., Garg S., Tao J., Brown D.J., Potma E.O., Jester J.V. (2019). Characterization of expressed human meibum using hyperspectral stimulated Raman scattering microscopy. Ocul. Surf..

[22] Bailey I.L., Bullimore M.A., Raasch T.W., Taylor H.R. (1991). Clinical grading and the effects of scaling. Investig. Ophthalmol. Vis. Sci..

[23] Paugh J.R., Tse J., Nguyen T., Sasai A., Chen E., De Jesus M.T., Kwan J., Nguyen A.L., Farid M., Garg S. (2020). Efficacy of the Fluorescein Tear Breakup Time Test in Dry Eye. Cornea.

[24] Lemp M.A., Crews L.A., Bron A.J., Foulks G.N., Sullivan B.D. (2012). Distribution of aqueous-deficient and evaporative dry eye in a clinic-based patient cohort: A retrospective study. Cornea.

[25] Paugh J.R., Nguyen T., Sasai A., Chen E., De Jesus M.T., Kwan J., Nguyen A.L., Farid M., Garg S., Jester J.V. (2022). The Efficacy of Clinical Tests to Diagnose Evaporative Dry Eye Disease Related to Meibomian Gland Dysfunction. J. Ophthalmol..

[26] Mackie I.A., Seal D.V. (1981). The questionably dry eye. Br. J. Ophthalmol..

[27] Tomlinson A., Bron A.J., Korb D.R., Amano S., Paugh J.R., Pearce E.I., Yee R., Yokoi N., Arita R., Dogru M. (2011). The International Workshop on Meibomian Gland Dysfunction: Report of the Diagnosis Subcommittee. Investig. Ophthalmol. Vis. Sci..

[28] Eom Y., Jun I., Jeon H.S., Lim D.H., Lee H., Hwang H.S., Chung S.H., Chung T.Y., Kim J.Y., Kim S.W. (2024). Re-Esterified Triglyceride omega-3 Fatty Acids in Dry Eye Disease With Meibomian Gland Dysfunction: A Randomized Clinical Trial. JAMA Ophthalmol..

[29] Foulks G.N., Bron A.J. (2003). Meibomian gland dysfunction: A clinical scheme for description, diagnosis, classification and grading. Ocul. Surf..

[30] Craig J.P., Blades K., Patel S. (1995). Tear lipid layer structure and stability following expression of the meibomian glands. Ophthal Phys. Opt..

[31] Arciniega J.C., Wojtowicz J.C., Mohamed E.M., McCulley J.P. (2011). Changes in the evaporation rate of tear film after digital expression of meibomian glands in patients with and without dry eye. Cornea.

[32] Korb D.R., Blackie C.A. (2013). Debridement-scaling: A new procedure that increases Meibomian gland function and reduces dry eye symptoms. Cornea.

[33] Ngo W., Caffery B., Srinivasan S., Jones L.W. (2015). Effect of Lid Debridement-Scaling in Sjogren Syndrome Dry Eye. Optom. Vis. Sci..

[34] Watson S.L., Jones L.W., Stapleton F., Hinds M., Ng A., Tan J., Alster Y., Bosworth C., Rafaeli O., DePuy V. (2023). Efficacy and safety of AZR-MD-001 selenium sulfide ophthalmic ointment in adults with meibomian gland dysfunction: A vehicle-controlled, randomized clinical trial. Ocul. Surf..

[35] Zhang Q., Zhang H., Qin G., Wu Y., Song Y., Yang L., Yu S., He X., Moore J.E., Moutari S. (2022). Impact of Diquafosol Ophthalmic Solution on Tear Film and Dry Eye Symptom in Type 2 Diabetic Dry Eye: A Pilot Study. J. Ocul. Pharmacol. Ther..

[36] Hjermstad M.J., Fayers P.M., Haugen D.F., Caraceni A., Hanks G.W., Loge J.H., Fainsinger R., Aass N., Kaasa S. (2011). European Palliative Care Research Collaborative. Studies comparing Numerical Rating Scales, Verbal Rating Scales, and Visual Analogue Scales for assessment of pain intensity in adults: A systematic literature review. J. Pain Symptom Manag..

[37] Karcioglu O., Topacoglu H., Dikme O., Dikme O. (2018). A systematic review of the pain scales in adults: Which to use?. Am. J. Emerg. Med..

[38] Korb D.R., Blackie C.A. (2011). Meibomian gland therapeutic expression: Quantifying the applied pressure and the limitation of resulting pain. Eye Contact Lens.

[39] Schanzlin D., Owen J.P., Klein S., Yeh T.N., Merchea M.M., Bullimore M.A. (2022). Efficacy of the Systane iLux Thermal Pulsation System for the Treatment of Meibomian Gland Dysfunction After 1 Week and 1 Month: A Prospective Study. Eye Contact Lens.

[40] Satitpitakul V., Ratanawongphaibul K., Kasetsuwan N., Reinprayoon U. (2019). Efficacy of azithromycin 1.5% eyedrops vs oral doxycycline in meibomian gland dysfunction: A randomized trial. Graefes Arch. Clin. Exp. Ophthalmol..

[41] Sakassegawa-Naves F.E., Ricci H.M.M., Moscovici B.K., Miyamoto D.A., Chiacchio B.B., Holzchuh R., Santo R.M., Hida R.Y. (2017). Tacrolimus Ointment for Refractory Posterior Blepharitis. Curr. Eye Res..

[42] Olafsson J., Lai X., Landsend E.C.S., Olafsson S., Parissi E., Utheim O.A., Raeder S., Badian R.A., Lagali N., Dartt D.A. (2021). TheraPearl Eye Mask and Blephasteam for the treatment of meibomian gland dysfunction: A randomized, comparative clinical trial. Sci. Rep..

[43] Efron N., Morgan P.B., Katsara S.S. (2000). Validation of grading scales for contact lens complications. Ophthal. Physiol. Opt..

[44] Wolffsohn J.S., Arita R., Chalmers R., Djalilian A., Dogru M., Dumbleton K., Gupta P.K., Karpecki P., Lazreg S., Pult H. (2017). TFOS DEWS II Diagnostic Methodology report. Ocul. Surf..

[45] Paugh J., Jiang J.R., Nguyen A.L. (2024). Core Outcome Parameters for Dry Eye Research: Meibomian Gland Secretion and Ocular Surfface Staining. Investig. Ophthalmol. Vis. Sci..

[46] Nelson J.D., Shimazaki J., Benitez-del-Castillo J.M., Craig J.P., McCulley J.P., Den S., Foulks G.N. (2011). The International Workshop on Meibomian Gland Dysfunction: Report of the Definition and Classification Subcommittee. Investig. Ophthalmol. Vis. Sci..

[47] McCulley J.P., Dougherty J.M., Deneau D.G. (1982). Classification of chronic blepharitis. Ophthalmology.

