# Supplementary material for: Meibomian Gland Outcome Measures in Dry Eye Treatment Trials
Source: J Clin Med. 2026 May 25;15(11):4093. doi: 10.3390/jcm15114093 (PMC13258495; doi:10.3390/jcm15114093)
Supplement: Supplementary file 1 [file jcm-15-04093-s001.zip › jcm-4275942-supplementary/jcm-4275942-supplementary.pdf]

Protocol for the Modified MGD Workshop Meibum Grading System (Version 1)

Shora Ansari, Jay Ruzhang Jiang, Jerry R. Paugh

April 7, 2026

Introduction and Rationale

Several systems for grading meibum have been reported in recent therapeutic trials, but the most prevalent is the Bron et al. system,[1], incorporated into the MGD Workshop system (**Table 4**, Management and Treatment report[2]) and that of Lane et al.[3] using the meibomian gland evaluator (MGE[4]. All systems have demonstrated efficacy in therapeutic trials.

A challenge of the Lane et al. system[3] is that it is physically difficult to assess three sectors of each lower eyelid, and to concurrently assess meibum quality from all 5 glands in each sector. It also may be unnecessary to evaluate 15 glands per eyelid as several studies assessing the central 8 glands have demonstrated response to treatment of MGD (e.g., Lee et al.,[5] Albietz and Schmid,[6] and Olafson et al.[7]).

In a recent therapeutic trial at our institution[8] we used a modified version of the MGD Workshop system (lower eyelid central 8 glands) and found the meibum score to be indicative of meibomian gland compromise and responsive to topical androgen therapy. While the central 8 glands were assessed, the investigators used a wooden handle cotton tipped applicator (CTA) to exert gentle pressure to about three gland orifices at a time, at a 45-degree angle to the eyelid margin, and the Bron scale to grade meibum quality: 0 – 4 scale for each gland, and consequent 0 – 32 scale for 8 glands summed.

A lower eyelid, central 8 glands assessed approach may be simpler to adopt in multicenter clinical trials and is more efficient by assessing fewer glands per eyelid. However, a limitation is that the system does not incorporate the standardized pressure of the MGE, used by Lane et al.[3].

This protocol, with rationale, is outlined below to standardize the modified MGD Workshop approach with a goal that further investigations for diagnostic test efficacy, repeatability and reproducibility can occur.

Protocol: Meibomian Gland Expressibility and Excreta Grading:

Physical Setup:

- A wooden handled CTA is used for the margin cleaning step and a fresh one for the expression proper. The wooden CTA is preferred to provide consistent pressure and angling during grading.
- A slit lamp biomicroscope set at 16X magnification and a just definite white light illumination

Eyelid Margin Preparation:

Clean the eyelid margin:

- A CTA is moistened using non-preserved saline
- The eyelid margin is gently pulled down toward the cheekbone, taking care not to press on the eyelid margin
- The moistened CTA is gently brushed along the eyelid margin in the central lower lid margin area to clean debris, makeup etc.; the subject should feel no more than a tickling sensation
- The same CTA is used to clean the fellow eyelid margin

Meibum Expression:

- A fresh, dry CTA is held approximately 1 inch (2.5 cm) from the cotton tip to allow consistent pressure application
- The CTA is held steady by placing fingers lightly along the temporal side of the face or along the bridge of the nose if evaluating the fellow eye
- Instruct the subject to look up, but to blink if they need to while looking upward
- Locate the 6:00 position of the lower eyelid, then go to the temporal or nasal side of the 6:00 position to begin grading
- At a 45 degree angle to the eyelid margin, press the CTA gently through the eyelashes at the margin proper so that the margin applanates (flattens) against the globe
    - The amount of pressure should be felt by the subject, but not painful
- Once pressure begins, roll the CTA downward slightly, to evert the margin toward the observer
- Hold gentle pressure for 5-10 seconds to express meibum (if available); often the meibum will express as a small "volcano" eruption if the gland orifice is partially restricted. If the meibum is clear and low viscosity, it will immediately express; e.g., in normals or in meibomian seborrhea subjects.
- Each placement and pressure should yield 3-4 meibomian orifices; record each individually on the 0 – 4 scale (see below for grading scale)
- Note that with thicker eyelids, the pressure against 3 orifices may induce expression from a neighboring gland
- It is helpful to have a scribe record the grades as they are observed
- Expressibility is a companion measure of meibomian gland function, assessed at the time of meibum grading for each gland. Whether meibum is expressed is a yes (1) or no (0) grading. If a grade for meibum can be determined, provide a "1" for expressibility at each pressure of the CTA as the gland orifices are assessed; record as the sum of all 8 glands assessed.

Figure 1: Applanation method to grade meibum quality

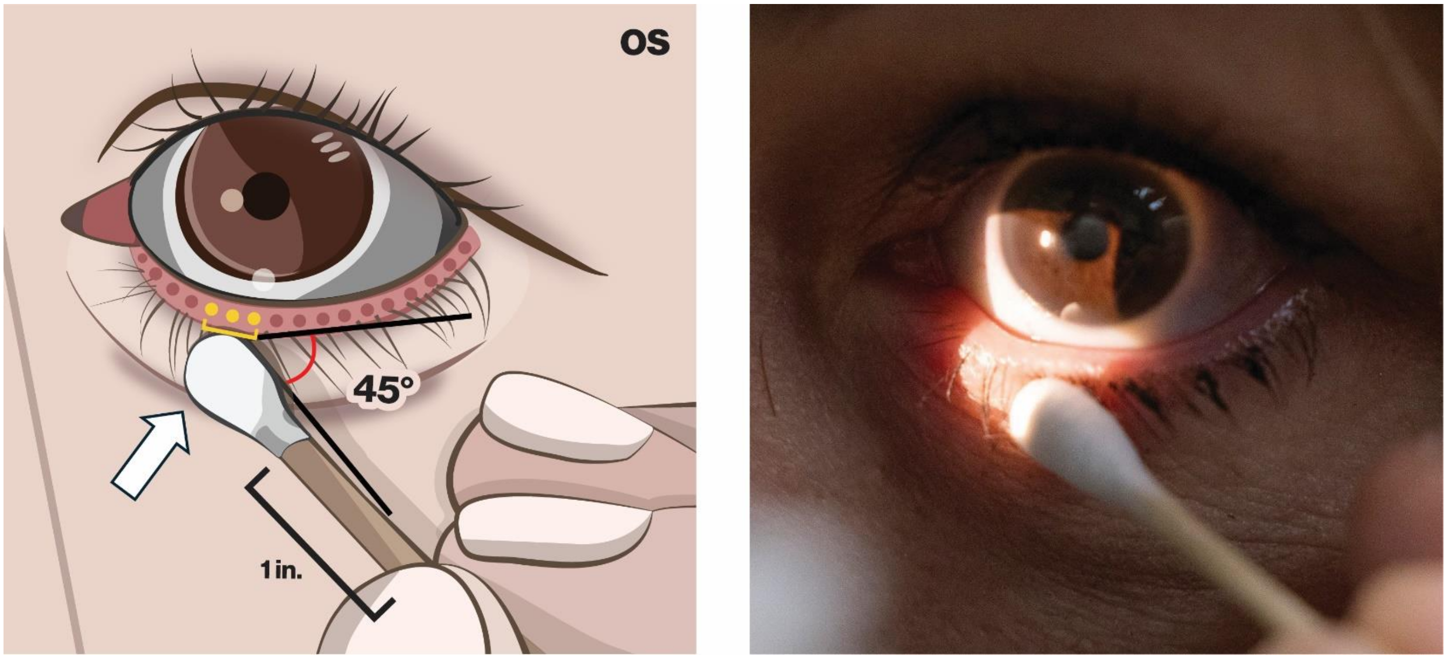

Meibum Grading: Background and Rationale:

1. The scale suggested in the MGD Workshop report (**Table 4**, Geerling et al.[2]) is related to the earlier reports of Bron and co-workers.[1, 9] and is a 0 – 3 scale for grading the quality of the expressed meibum. A further refinement of the total scale is helpful, to add a “4” for glands that do not exhibit meibum expression, either due to a capped gland or a missing gland[10, 11]. Using a score of 0 – 4 for each gland provides a total scale for each eyelid of 0 – 32.
2. A second change to the MGD Workshop scale is to use finer scale increments for each gland meibum grade rather than integers. Bailey et al.[12] have demonstrated that finer scale increments allow greater ability to detect change compared to an integer scale when using a clinical scale. Herein are suggested 0.5 scale unit increments; e.g., 0, 0.5, 1.0, 1.5, 2.0, 2.5, etc. to allow for meibum grades between traditional integer levels. Use of finer scale increments appears achievable and desirable[13], and produces a semi-continuous scale, useful for detecting change and in statistical analysis.
3. A scribe to record the meibum grade as the procedure occurs will help the reliability and efficiency of the assessment.

Meibum and Expressibility Grading: protocol:

Meibum:

- o Slit lamp set at 16X magnification, with definite, but not extreme white light, medium width beam
- o Use a wooden handle cotton tipped applicator (CTA), of about 2+ inches in length for margin cleaning and for applanation and grading.
- o Both Right and Left eyes, lower eyelids, can be examined similarly (left is easier for a right-handed person and vice versa)
- o Find the 6:00 position of the cornea as the central point
- o Gently brush the eyelid margin with a saline-moistened CTA across the central 8 glands to remove cellular debris, etc. This makes the secretions easier to visualize.
- o Gently apply a second, dry CTA “end on” at a 45 degree angle to the eyelid, through the eyelashes, to applanate against the globe.
    - o Tilt down slightly, everting the margin toward the observer to maximize the observation of grading.
    - o This allows approximately 3 orifices to be graded concurrently.
    - o See **Figure 1**
- Maintain gentle pressure (where subject does not report pain) for 5 – 10 seconds until excreta is observed
- Grade each orifice with a value of 0 – 4 in 0.5 scale unit increments; **see scale below**.
    - o Observer or scribe records on a diagram on the case report form (CRF)
- o Shift the CTA two more times to the next groups of 3 glands on the same lower eyelid; ignore those beyond the central 8 glands. The method is to bracket the 6:00 position to obtain 8 glands’ excreta values.
- o Add up individual scores (0 – 4) for 8 glands to provide a summed value (0 – 32 total scale)

Expressibility:

- For each of the 8 orifices, record a value of “1” if liquid meibum is observed, or “0” if none is observed.
- Sum the values for 8 gland orifices; the total is the expressibility score for each eyelid.

The Meibum Grading Scale is an “opacity – viscosity” scale, similar to Mathers et al.,1991[14]

Grading:

- 0 = clear, low viscosity, easily expressed, may have a few particles
  0.5 = partially opaque, low viscosity, easily expressed, may have a few particles
- 1.0 = opaque, low viscosity, obscures margin details
  1.5 = opaque, slightly elevated viscosity (greater than 1.0, but less than 2.0)
- 2.0 = opaque, increased viscosity, may extrude gel-like globules
  2.5 = opaque, not completely inspissated
- 3.0 = opaque, inspissated, like toothpaste
  3.5 = opaque, secretions retain form after expression (e.g., pouting[1])
- 4.0 = no excreta visible (due to blocked or missing gland)

Recording: a diagram with 8 boxes for each of expressibility (0 or 1) and 0 – 4 for meibum quality can be developed

Scoring:

1. sum expressibility as 0 – 8 for each eyelid
2. sum meibum quality as 0 – 4 scores for each of 8 glands; total scale 0 – 32 per eyelid

References

[1] Bron AJ, Benjamin L, Snibson GR. Meibomian gland disease. Classification and grading of lid changes. Eye. 1991;5:395-411.
[2] Geerling G, Tauber, J., Baudouin, C., Goto, E., Matsumoto, Y., O'Brien, T., Rolando, M., Tsubota, K., Nichols, K.K. The International Workshop on Meibomian Gland Dysfunction: Report of the Subcommittee on Management and Treatment of Meibomian Gland Dysfunction. Invest Ophthalmol Vis Sci. 2011;52:2050-64.
[3] Lane SS, DuBiner HB, Epstein RJ, Ernest PH, Greiner JV, Hardten DR, et al. A new system, the LipiFlow, for the treatment of meibomian gland dysfunction. Cornea. 2012;31:396-404.
[4] Blackie CA, Korb DR. The diurnal secretory characteristics of individual meibomian glands. Cornea. 2010;29:34-8.
[5] Lee H, Chung B, Kim KS, Seo KY, Choi BJ, Kim TI. Effects of topical loteprednol etabonate on tear cytokines and clinical outcomes in moderate and severe meibomian gland dysfunction: randomized clinical trial. Am J Ophthalmol. 2014;158:1172-83 e1.
[6] Albietz JM, Schmid KL. Intense pulsed light treatment and meibomian gland expression for moderate to advanced meibomian gland dysfunction. Clin Exp Optom. 2018;101:23-33.
[7] Olafsson J, Lai X, Landsend ECS, Olafsson S, Parissi E, Utheim OA, et al. TheraPearl Eye Mask and Blephasteam for the treatment of meibomian gland dysfunction: a randomized, comparative clinical trial. Sci Rep. 2021;11:22386.
[8] Jiang JR, Khankan R, Ridder WH, 3rd, Nguyen AL, Paugh JR. A pilot randomized controlled trial of topical androgen treatment in dry eye. Ocul Surf. 2025;38:56-63.

[9] Foulks GN, Bron, A.J. Meibomian gland dysfunction: a clinical scheme for description, diagnosis, classification and grading. The Ocular Surface. 2003;1:107-26.
[10] Wojtowicz JC, Butovich I, Uchiyama E, Aronowicz J, Agee S, McCulley JP. Pilot, prospective, randomized, double-masked, placebo-controlled clinical trial of an omega-3 supplement for dry eye. Cornea. 2011;30:308-14.
[11] Fadlallah A, Rami HE, Fahd D, Dunia I, Bejjani R, Chlela E, et al. Azithromycin 1.5% ophthalmic solution: efficacy and treatment modalities in chronic blepharitis. Arq Bras Oftalmol. 2012;75:178-82.
[12] Bailey IL, Bullimore, M.A., Raasch, T.W., Taylor, H.R. Clinical grading and the effects of scaling. Invest Ophthalmol Vis Sci. 1991;32:422-32.
[13] Efron N, Morgan, P.B., Katsara, S.S. Validation of grading scales for contact lens complications. Ophthal Physiol Opt. 2000;21:17-29.
[14] Mathers WD, Shields, W.J., Sachdev, M.S., Petroll, W.M., Jester JV. Meibomian gland dysfunction in chronic blepharitis. Cornea. 1991;10:277-85.
