# Supplementary material for: Meibomian Gland Outcome Measures in Dry Eye Treatment Trials
Source: J Clin Med. 2026 May 25;15(11):4093. doi: 10.3390/jcm15114093 (PMC13258495; doi:10.3390/jcm15114093)
Supplement: Supplementary file 1 [file jcm-15-04093-s001.zip › jcm-4275942-supplementary/Retrospective Chart Review Data, from Paugh et al., 2019, 4-7-26.pdf]

Retrospective Chart Review Data, from: Paugh et al., Characterization of expressed human meibum using hyperspectral stimulated Raman scattering spectroscopy, The Ocular Surface, 2019; 151 - 159.

Normals

Subject #, Sex, Age	Opacity Grade	Viscosity Grade	Bron Grade	Examiner Description
21, F, 54	0.5	0.8	0.9	clear, particles
29, M, 73	1.0	0.8	0.9	clear, particles
31, M, 52	0.3	1.0	0.7	clear mostly
33, F, 30	0.7	1.0	0.9	clear, particles
36, F, 24	1.0	1.0	0.9	No description
37, F 48	1.1	1.5	1.7	clear, viscous, particles
38, F, 22	0.5	0.5	0.6	clearish
39, F, 22	0.7	1.0	0.9	clear, particles
40, F, 25	0.9	0.7	1.2	clear, particles
41, M, 24	1.8	1.4	1.9	nearly opaque
45, F, 48	1.8	1.5	1.9	clear, larger particles
46, M, 23	1.2	1.5	2.2	clear, particles
50, F, 65	0.8	1.0	0.9	clear, particles
51, F, 27	0.7	0.6	0.9	clear, particles
53, F, 47	2.0	1.5	1.9	nearly opaque, viscous, particles
55, F, 29	1.2	1.0	1.2	clear, some more viscous
58, F, 23	1.0	1.0	0.9	clear to yellow, particles
61, M, 29	1.2	1.0	1.0	yellow, mostly opaque, particles
62, M, 28	1.2	1.0	1.0	cloudy to yellow, mostly opaque, particles
63, F, 22	1.9	1.5	2.0	opaque, yellow
64, F, 23	0.9	1.0	0.9	clear, particles
65, F, 28	0.7	1.0	0.9	clear, particles
66, F, 57	0.9	0.8	0.9	clear, particles
68, M, 23	1.2	1.0	1.0	opaque, small particles
72, F, 62	0.4	0.7	0.7	clear mostly
74, F, 34	1.0	1.0	1.0	opaque, very small paricles
76, F, 26	0.7	0.7	0.9	clear to slightly yellow, particles
78, M, 38	0.8	0.8	0.9	clear, particles
79, F, 27	1.2	1.0	1.5	some opacity, particles
80, F, 30	0.8	1.0	0.9	clear, particles
83, M, 49	0.8	0.8	1.0	both clear and opaque, particles
**Mean**	**1.0**	**1.0**	**1.1**	
**SD**	**0.4**	**0.3**	**0.4**	

Aqueous Deficients

Subject #, Sex, Age	Opacity Grade	Viscosity Grade	Bron Grade	Examiner Description
25, M, 73	1.8	1.5	1.8	No description
27, F, 66	2.0	2.5	2.5	clear, particles and opaque, inspissated
34, F, 27	1.0	1.0	1.2	clear, many particles
94, F, 42	0.6	0.5	0.9	clear and opaque, particles
112, M, 36	0.5	0.7	0.9	No description
129, M, 74	1.8	2.0	2.0	opaque, particles
137, F, 71	NA	NA	1.0	No description
142, F, 63	NA	NA	1.0	No description
1, F, 82	NA	NA	2.0	No description
6, F, 67	NA	NA	2.0	opaque, particles
11, F, 69	NA	NA	1.0	opaque
13, F, 73	1.0	0.5	1.9	No description
**Mean**	**1.2**	**1.2**	**1.5**	
**SD**	**0.6**	**0.8**	**0.6**	

MGD

Subject #, Sex, Age	Opacity Grade	Viscosity Grade	Bron Grade	Examiner Description
23, M, 26	2.0	1.8	1.9	No description
24, F, 57	2.0	1.8	1.9	opaque, nearly inspissated, particles
26, M, 83	1.0	0.8	1.8	No description
28, M, 74	1.9	1.8	2.0	opaque, yellow
30, F, 66	1.5	1.2	1.8	opaque, particles
32, F, 24	1.9	2.1	2.0	yellow, viscous, some inspissated
35, M, 29	0.5	0.6	0.5	clear, particles
42, F, 64	1.0	0.9	0.9	clear, particles
43, M, 22	0.0	0.0	0.2	clear, particles
44, F, 74	2.0	1.8	1.9	nearly opaque, small particles
47, M, 73	2.0	2.0	2.2	opaque, particles
52, M, 44	2.2	2.0	2.0	opaque, small particles
54, M, 67	0.7	0.7	0.9	clear, particles
57, F, 54	2.0	2.0	2.0	opaque, yellow
59, F, 69	2.0	1.5	2.0	some viscous, yellow, particles
71, M, 69	0.7	0.8	0.9	clear, particles
73, M, 68	1.2	1.4	1.2	opaque, particles
75, F, 43	2.0	1.5	1.2	No description
81, F, 50	2.8	2.8	2.7	mostly inspissated, some clear oil
82, M, 53	1.5	1.0	1.0	clear and opaque, particles
84, M, 66	0.8	1.0	0.9	clear, particles
86, M, 76	2.0	1.8	2.0	opaque, globules granular
91, F, 52	0.8	0.9	0.9	clear and opaque, particles
96, F, 71	0.7	0.7	0.9	clear, particles
98, M, 64	0.5	0.5	0.9	clear, particles
106, M, 30	0.8	0.7	0.9	clear, particles
107, F, 58	0.5	0.5	0.9	clear, particles
108, F, 37	1.5	1.0	1.0	opaque, some inspissated
109, F, 25	1.0	0.6	1.0	opaque
113, F, 58	2.0	2.0	2.5	opaque, some inspissated
117, F, 65	2	2	2	clear, particles, and inspissated
**Mean**	**1.4**	**1.3**	**1.4**	
**SD**	**0.7**	**0.7**	**0.6**	

Retrospective Chart Data, Not previously published: from the study of Paugh, JR, et al., "Characterization of expressed human meibum using hyperspectral stimulated Raman scattering pectroscopy": The Ocular Surface, 2019; 151 - 159.

Grading Scales and Descriptions: opacity, viscosity scales graded using 0.1 scale unit increments; Bron scale in integer increments

Opacity (cloudiness in original CRF): 0 = clear, 1 = partial obscuration of margin detail, 2 = mostly obscures margin detail, 3 = opaque

Viscosity: 0 = olive oil, 1 = slow spreading, 2 = very slow spreading, like corn syrup, 3 = toothpaste

Bron scale (approximate, as in MGD Workshop Table 4): 0 = clear, normal, 1 = cloudy, 2 = cloudy with particles, 3 = inspissated, like toothpaste

Grading:

1. data are from one eye only; the worst eye according to NEI grading for cornea
2. Single investigator made assessment (JRP)
3. wooden handle cotton tipped applicator pressed tangentially along lower eyelid margin - applied for 5 - 10 seconds
4. white light, 10X magnification
5. grades and descriptions stemming from an average of the entire lower eyelid
